# Robustness and Plasticity of Metabolic Pathway Flux among Uropathogenic Isolates of *Pseudomonas aeruginosa*

**DOI:** 10.1371/journal.pone.0088368

**Published:** 2014-04-07

**Authors:** Antje Berger, Katrin Dohnt, Petra Tielen, Dieter Jahn, Judith Becker, Christoph Wittmann

**Affiliations:** 1 Institute of Biochemical Engineering, Technische Universität Braunschweig, Braunschweig, Germany; 2 Institute of Microbiology, Technische Universität Braunschweig, Braunschweig, Germany; 3 Institute of Systems Biotechnology, Saarland University, Saarbrücken, Germany; Virginia Commonwealth University, United States of America

## Abstract

*Pseudomonas aeruginosa* is a human pathogen that frequently causes urinary tract and catheter-associated urinary tract infections. Here, using ^13^C-metabolic flux analysis, we conducted quantitative analysis of metabolic fluxes in the model strain *P. aeruginosa* PAO1 and 17 clinical isolates. All *P. aeruginosa* strains catabolized glucose through the Entner-Doudoroff pathway with fully respiratory metabolism and no overflow. Together with other NADPH supplying reactions, this high-flux pathway provided by far more NADPH than needed for anabolism: a benefit for the pathogen to counteract oxidative stress imposed by the host. *P. aeruginosa* recruited the pentose phosphate pathway exclusively for biosynthesis. In contrast to glycolytic metabolism, which was conserved among all isolates, the flux through pyruvate metabolism, the tricarboxylic acid cycle, and the glyoxylate shunt was highly variable, likely caused by adaptive processes in individual strains during infection. This aspect of metabolism was niche-specific with respect to the corresponding flux because strains isolated from the urinary tract clustered separately from those originating from catheter-associated infections. Interestingly, most glucose-grown strains exhibited significant flux through the glyoxylate shunt. Projection into the theoretical flux space, which was computed using elementary flux-mode analysis, indicated that *P. aeruginosa* metabolism is optimized for efficient growth and exhibits significant potential for increasing NADPH supply to drive oxidative stress response.

## Introduction

*Pseudomonas aeruginosa* is a metabolically versatile bacterium that resides in a wide range of biotic and abiotic habitats and is a human pathogen that causes numerous acute and opportunistic infections [1]. The clinical spectrum of *P. aeruginosa* infections includes wound and urinary tract infections, meningitis, and necrotizing pneumonia [2]. In particular, urinary tract infections and catheter-associated urinary tract infections are the most common bacterial infections in clinical practice [3], [4] and pose a severe health threat with more than one million hospitalizations annually [5].

Research on *P. aeruginosa* has focused its virulence [1], resistance [6], and adaptation [7] as well as therapeutic strategies [8]. Microevolution through resistance-mediating mutations in the bacterium's resistome involves a large subset of its genetic repertoire and a complex network of metabolic pathways that mediate adaptive resistance and adaptive metabolism [9]–[11]. Therefore, a systems-level understanding of the network that drives the pathogenesis of *P. aeruginosa* is important for devising specific control strategies [1]. In particular, ^13^C-metabolic flux analysis (fluxomics) detects common and specific pathways employed by pathogens and identifies candidate pathways as targets for therapy [12], [13].

This network-wide approach provides information on the activities of central enzymes and pathways most directly linked to phenotype [14]. However, to our knowledge, such analyses of *P. aeruginosa* have not been published. Here, we investigated the laboratory strain *P. aeruginosa* PAO1 at the level of carbon fluxes by using ^13^C-metabolic flux analysis that combined isotopic tracer experiments with mass spectrometric labeling analysis and stoichiometric and isotopomer balancing for flux calculation [15]. This was extended to a collection of 17 *P. aeruginosa* clinical isolates from patients with urinary tract infections and catheter-associated urinary tract infections. These strains are genetically diverse, differ from strains that cause chronic lung infections in patients with cystic fibrosis, and exhibit heterogeneous production of virulence factors *in vitro* [16].

## Materials and Methods

### Bacteria

The model strain, *P. aeruginosa* PAO1 served as a reference [17]. Uropathogenic *P. aeruginosa* isolates from patients with direct urinary tract infections included the strains MH06u, MH09u, RN12u, RN13u, MH16u, MH17u, MH26u, and MH29u. Isolates from patients with catheter-associated urinary tract infections included the strains MH15c, MH25c, MH33c, MH34c, MH36c, MH37c, MH39c, MH56c, and MH57c [16]. *Corynebacterium glutamicum* ATCC 13032 and *P. putida* KT2440 were acquired from the German Collection of Strains and Cell Cultures (DSMZ, Braunschweig, Germany). All strains were stored in 10% (w/v) glycerol at −80°C.

### Culture media

Starter cultures were grown in complex LB medium containing 5 g·L^−1^ yeast extract (Becton, Dickinson and Company, Sparks, MD, USA), 10 g·L^−1^ peptone (Becton, Dickinson and Company) and 10 g·L^−1^ NaCl. For the second and main cultures, a minimal medium developed during the study ensured balanced growth of all strains, an important prerequisite for the ^13^C-flux approach [15]. The osmolality of this medium was 500 mosmol·kg^−1^, which reflects that of human urine [18]. The medium contained the following (per liter): 2.5 g glucose, 13.0 g KH_2_PO_4_×2 H_2_O, 20.6 g K_2_HPO_4_, 0.49 g MgSO_4_×7 H_2_O, 2.5 g NH_4_Cl, 1.41 g Na_2_SO_4_, 0.085 g CaCl_2_×2 H_2_O, 1.2 mg FeSO_4_×7 H_2_O, and 25 mg 3,4-dihydroxybenzoate. For ^13^C-flux experiments, naturally labeled glucose was replaced with 99% [1-^13^C] glucose (Cambridge Isotope Laboratories, Inc., Andover, MA, USA).

### Culture conditions

Cultures were shaken at 1,050 rpm and 37°C in 10 mL deep-well plates (HJ Bioanalytik, Mönchengladbach, Germany) with a working volume of 1.4 mL (Titramax 1000, Heidolph Instruments, Schwabach, Germany). Starter cultures were prepared by inoculating LB medium with 20 µL of a glycerol stock. After 6 h of incubation, 50 µL of cell suspension was transferred to a second culture in minimal medium. Subsequently, exponentially growing cells (50 µL) were used as inoculum for main cultures, and strains were grown in 30 mL of minimal medium in 300 mL baffled shake flasks. These were incubated at 37°C with shaking at 200 rpm on an orbital shaker (Aquatron, Infors AG, Switzerland). Dissolved oxygen was monitored online using shake flasks with integrated sensor spots (PreSens SFR, PreSens Precision Sensing GmbH, Regensburg, Germany). The sensor device was installed in the orbital shaker as described previously [19]. In cultures incubated with ^13^C-tracer, the inoculum level was always kept below 1% of the final sampled cell concentration to exclude potential interference of a differently labeled inoculum on subsequent calculation of flux [15].

### Quantification of cell concentration

Cell growth was monitored spectrophotometrically at 578 nm (OD_578_) (Libra S11, Biochrome, Cambridge, UK). Cell dry weight (CDW) was determined as follows: Dried plastic tubes were filled with 20 mL cell suspension, and cells were harvested by centrifugation (25,800× *g*, 4°C, 10 min); subsequently, pellets were washed twice with deionized water, and then dried to a constant weight.

### Quantification of substrates and products

The concentration of glucose was determined from filtered supernatants (Minisart, 0.2 µm, Sartorius, Göttingen, Germany) using a YSI 2700 Select biochemical analyzer (Kreienbaum, Langenfeld, Germany). The analysis of organic acids (oxalic acid, citric acid, α-ketoglutaric acid, gluconic acid, glyoxylic acid, pyruvic acid, succinic acid, glycolic acid, lactic acid, fumaric acid, acetic acid, propanoic acid, and butyric acid) and amino acids in the culture supernatant was conducted using high-pressure liquid chromatography [20]. The limit of detection was below 10 µM for organic acids and below 1 µM for amino acids, respectively.

### Enzyme assays

Cultures (10 mL) were harvested by centrifugation (25,800× *g*, 4°C, 5 min), washed with 100 mM Tris-HCl (pH 7.5) containing 10 mM MgCl_2_ and 0.75 mM dithiothreitol, and then resuspended in the same buffer. The cells were then mechanically disrupted (100 µm silica glass beads, 2×20 s, 6.0 m·s^−1^, FastPrep-24, MP Biomedicals, Eschwege, Germany). Total protein concentration of the obtained cell extract was determined using Roti-Quant (Carl Roth GmbH, Karlsruhe, Germany). Phosphofructokinase activity was determined spectrophotometrically at 340 nm [21]. The reaction mixture contained 100 mM Tris-HCl (pH 8.0), 0.5 U·mL^−1^ aldolase, 1 U·mL^−1^ triosephosphate isomerase, 0.5 U·ml^−1^ glycerol-phosphate dehydrogenase, 5 mM MgCl_2_, 0.25 mM NADH, 0.1 mM ATP, and 4 mM fructose 6-phosphate. The activity of isocitrate lyase was determined as described previously [22]. Reactions were initiated by adding substrate, and reactions without substrate served as negative controls. One unit (U) of activity represents the conversion of 1 µmol substrate per minute.

### Mass isotopomer labeling analysis of proteinogenic amino acids

Cells (0.5 mg CDW) were harvested in the mid-exponential growth phase by centrifugation (3 min, 16,000× *g*, 4°C, Biofuge Fresco, Heraeus, Hanau, Germany), washed twice with deionized water, and incubated with 30 µL 6 M HCl for 24 h at 100°C. The obtained hydrolysate was filtered (0.2 µm, Ultrafree MC, Millipore, Bedford, MA, USA) and then lyophilized. The proteinogenic amino acids in the lyophilisate were dissolved in 50 µL *N*, *N*-dimethylformamide containing 1% (v/v) pyridine and then derivatized at 80°C for 30 min with 50 µL N-methyl-*t*-butyldimethylsilyl-trifluoroacetamide (Macherey-Nagel, Düren, Germany). Mass isotopomer distributions of selected ion clusters of the analytes were then quantified using gas chromatography-mass spectrometry (GC-MS) (HP6890, M 5973, Agilent Technologies, Waldbronn, Germany) as described earlier [23]. All samples were first measured in scan mode to exclude isobaric overlay, and were analyzed in triplicate by selective ion monitoring to determine relative fractions of the mass isotopomers of interest [15].

### Metabolic reaction network and flux calculation

A large-scale model of the central metabolism of *P. aeruginosa* was assembled according to the genome-scale model of *P. aeruginosa* PAO1 [24], KEGG database [25], and the *Pseudomonas* genome database [26]. The network compromised the Entner-Doudoroff pathway (EDP), incomplete Embden-Meyerhof-Parnas pathway lacking phosphofructokinase (EMPP), pentose phosphate pathway (PPP), gluconeogenesis, reactions of pyruvate carboxylase, PEP carboxylase, PEP carboxykinase, malic enzyme, tricarboxylic acid (TCA) cycle, glyoxylate pathway, and anabolic pathways generating biomass. The chemical composition of *P. aeruginosa* cells was adapted from that of closely related *Pseudomonas* species [27], [28]. Because *P. aeruginosa* uniquely synthesizes the capsular polysaccharide alginate [29], the cellular content of this polymer was measured (Table S1, [30]), and the corresponding precursor demand was considered for all strains. The calculation of metabolic flux through the network was performed using OpenFLUX software [31]. For each strain, the mass isotopomer distributions of the derivatized amino acid residues [M-57] of alanine (*m/z* 260), glycine (*m/z* 246), valine (*m/z* 288), serine (*m/z* 390), threonine (*m/z* 404), phenylalanine (*m/z* 336), aspartate (*m/z* 418), glutamate (*m/z* 432), tyrosine (*m/z* 466), and the [M-85] fragment of serine (*m/z* 362) were used as input after correction for natural isotopes [32]. Multiple flux estimations using statistically varied starting values for free flux parameters confirmed the identification of a global minimum. For all flux data, 95% confidence intervals were calculated using a Monte Carlo approach [33].

### Elementary flux mode analysis

The metabolic network of *P. aeruginosa* was investigated using in silico pathway analysis involving the computation of elementary flux modes. The network topology agreed with that of the related species *P. putida* [34]. The energy demand for polymerization of building blocks and biomass assembly was based on the genome model of *P. aeruginosa* PAO1 [24]. Elementary flux modes were calculated as described previously [35]. Evaluation of the modes was carried out using Excel (MS Office, Windows, 2007) and provided relative pathway flux and yields for each of the modes [34], [35].

### Statistical analysis

Hierarchical clustering analysis (HCA) and principal component analysis (PCA) were conducted using MATLAB (Version R2011b, The MathWorks). PCA was used to convert the set of ^13^C-labeling data to a set of linearly uncorrelated variables, i.e., the principal components [36]. HCA was carried out using the complete linkage, the Euclidean distance, as a measure of proximity of experimental data to inspect strain similarity [37].

## Results

### Quantitative metabolism of *P. aeruginosa* PAO1 and the clinical isolates

*P. aeruginosa* PAO1 consumed glucose from an early time point (Figure S1), and its growth rate was approximately 0.91/h, corresponding to a doubling time of 45 min (Table 1). Biomass and CO_2_ were considered to be the only products formed, because secreted by-products were not detected. Note that the strain grew fully aerobically as verified by on-line monitoring of the dissolved oxygen level (Figure S2). Next, we analyzed *P. aeruginosa* isolates from urinary tract infections (8 strains) and catheter-associated urinary tract infections (9 strains). All individual growth profiles are presented in the Supporting Information (Figure S3). The strains showed high variability in growth (Table 1).

**Table 1:** Kinetics and stoichiometry of glucose-grown *P. aeruginosa* PAO1 and uropathogenic *P. aeruginosa* isolates obtained from patients with catheter-associated urinary tract infection or urinary tract infections.

Strain	Maximum specific growth rate (h^−1^)	Biomass yield (g_CDW_ g_Glucose_^−1^)	Glucose uptake rate (mmol g_CDW_^−1^ h^−1^)
PAO1	0.91±0.02	0.53±0.01	9.52±0.21
MH15c	0.35±0.01	0.36±0.01	5.37±0.29
MH25c	0.67±0.04	0.45±0.00	8.28±0.58
MH33c	0.91±0.02	0.51±0.01	9.87±0.17
MH34c	0.88±0.03	0.53±0.01	9.30±0.16
MH36c	0.70±0.02	0.44±0.01	8.92±0.35
MH37c	0.62±0.02	0.43±0.01	8.08±0.40
MH39c	0.60±0.01	0.41±0.01	8.04±0.05
MH56c	0.93±0.01	0.51±0.01	10.16±0.07
MH57c	0.85±0.02	0.39±0.00	12.22±0.28
MH06u	0.81±0.04	0.49±0.01	9.31±0.58
MH09u	0.74±0.03	0.49±0.02	8.33±0.11
RN12u	0.67±0.02	0.47±0.00	7.94±0.25
RN13u	0.82±0.03	0.48±0.02	9.49±0.40
MH16u	0.52±0.01	0.51±0.02	5.75±0.10
MH17u	0.59±0.01	0.50±0.02	6.66±0.25
MH26u	0.65±0.02	0.44±0.02	8.24±0.17
MH29u	0.82±0.03	0.53±0.00	8.65±0.33

The corresponding cultivation profiles for all strains are provided in Supporting Information (Figure S3). Values indicate means and standard deviations of three biological replicates. By-products in the supernatants were not detected; concentrations were below the detection limit (1 µM for amino acids and 10 µM for organic acids).

The efficiency for recruiting glucose for anabolism, i.e., the biomass yield, differed by more than a factor of 1.5. Strain MH15c produced the lowest biomass yield, which was approximately 65% of the value of the two most efficient strains, MH34c and MH29u, which yielded a biomass 0.53 g·g^−1^. This approached the theoretical optimum (0.54 g·g^−1^), a value derived by in silico pathway simulation. Briefly, the corresponding simulation for the carbon core network of *P. aeruginosa* provided 13,138 elementary modes, each representing a theoretical metabolic state of the cell. Together, all modes span the feasible flux space of the organism and contain the optimum growth mode with maximum possible yield [35]. Differences between growth kinetics of the strains were even larger. Whereas certain strains grew rather slowly and exhibited doubling times of about 120 min, others duplicated within only 40 min. Similarly, specific uptake rates for glucose differed significantly.

### Glucose catabolism by *P. aeruginosa* PAO1

**Figure 1:**
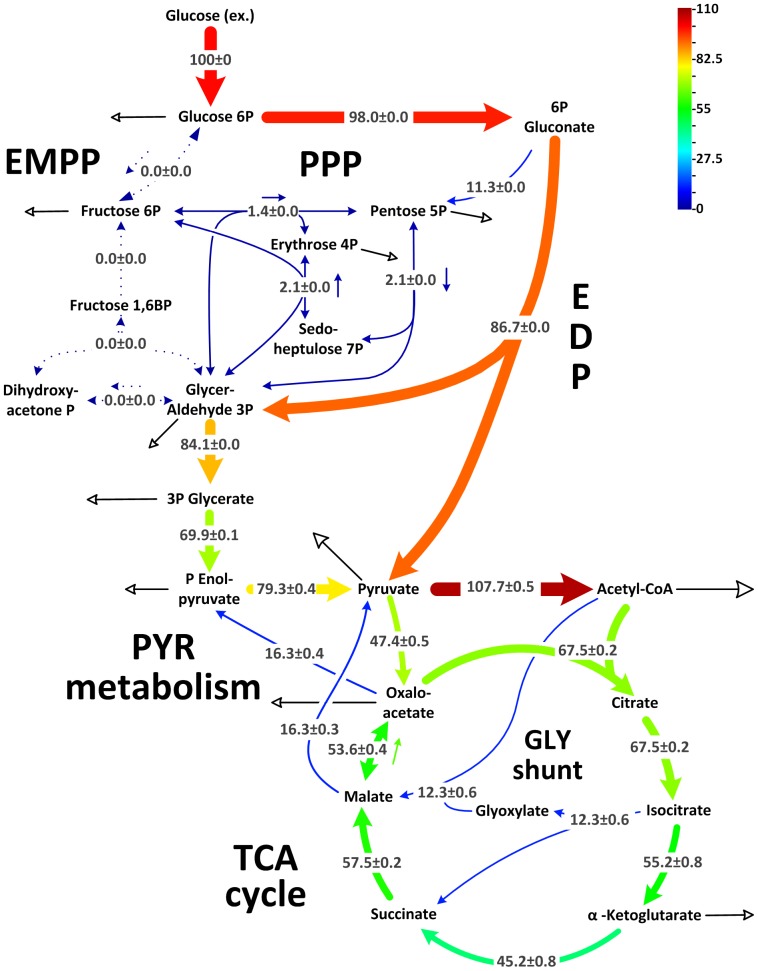
In vivo carbon flux distribution in the central metabolism of *P. aeruginosa* PAO1. Flux is expressed as a molar percentage of the specific glucose uptake rate of 9.5^−1^ h^−1^. Open arrows indicate the flux toward biomass. For reversible reactions, the direction of the net flux is indicated by a dashed arrow. The errors given for each flux reflect the corresponding 90% confidence intervals. The full flux data set is presented in Supporting Information. Metabolic and isotopic steady states were ensured by constant stoichiometry, kinetics, and the constant ^13^C-labeling patterns of recruited metabolites during cultivation (see Figure S1, Figure S5). The abbreviations are as follows: Entner-Doudoroff pathway (EDP), Embden-Meyerhof-Parnas pathway (EMPP), pentose phosphate pathway (PPP), glyoxylate (Gly) shunt, tricarboxylic acid (TCA) cycle, and pyruvate metabolism.

*P. aeruginosa* PAO1 was grown on [1-^13^C] glucose to estimate metabolic flux and exhibited metabolic and isotopic steady state growth (see Supporting Information). This fulfilled an important prerequisite for the ^13^C-flux approach [15]. The simulated labeling data, corresponding to the optimal fit, agreed closely with experimental values (Table S2) and indicated high consistency of the flux values. Glucose was mainly consumed through the EDP (Figure 1). The flux through 6-phosphogluconate dehydrogenase, which catalyzes the first reaction of the oxidative PPP, was low. The PPP exclusively served for anabolic purposes. Both, oxidative and non-oxidative PPP, contributed to supply of ribose 5-phosphate, erythrose 4-phosphate, and fructose 6-phosphate for anabolism. EMPP activity was undetectable, likely caused by lack of cytoplasmic phosphofructokinase activity (<0.01 mU·mg^−1^). Accordingly, *P. aeruginosa* PAO1 catabolized glucose only through the EDP to subsequent glycolytic steps and further to pyruvate.

### Pyruvate metabolism, anaplerosis, and the TCA cycle in *P. aeruginosa* PAO1

**Figure 2:**
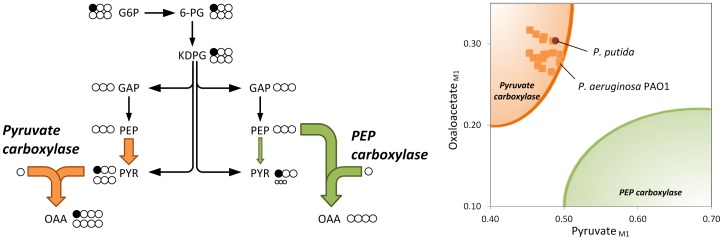
Resolution of anaplerotic flux in [1-^13^C]-glucose-grown uropathogenic *P. aeruginosa*, *P. aeruginosa* PAO1, and *P. putida*. For exclusive use of the ED pathway for glucose catabolism, the contribution of PEP carboxylase resulted in low ^13^C-enrichment of oxaloacetate (deduced from aspartate), whereas the ^13^C accumulated in pyruvate (alanine), which creates an excess of its single-labeled (M1) mass isotopomer (green region). In contrast, the action of pyruvate carboxylase reduced and increased the levels of pyruvate and oxaloacetate (orange region), respectively. This allowed for discrimination between these enzymes according to the combined labeling of the two molecules. Prior to inspection, the ^13^C-labelling data were corrected for the contribution of natural isotopes [32].

The activity of a functional PEP carboxylase (anaplerotic reaction from phosphoenolpyruvate to oxaloacetate) could not be confirmed. During growth on [1-^13^C] glucose, the combination of ^13^C enrichment in pyruvate and oxaloacetate can be used to discriminative between PEP and pyruvate carboxylase (Figure 2). The weak signal generated by the singly labeled pyruvate mass isotopomer (M1), i.e., the low ^13^C-enrichment in the molecule, together with a relatively high value for the corresponding M1 isotopomer of oxaloacetate, matched the expected pattern for pyruvate carboxylase. In contrast, it differed significantly from possible combinations for pyruvate and oxaloacetate, which would result from PEP carboxylase. Thus, pyruvate was the source of anaplerotic oxaloacetate.

*P. putida* expresses pyruvate carboxylase [27] and shows the same pattern (Figure 2). On flux level, decarboxylating phosphoenolpyruvate carboxykinase and malic enzyme returned carbon to the glycolytic pools (Figure 1). Accordingly, malic enzyme powered the pyruvate shunt (malate conversion to pyruvate by malic enzyme and further to oxaloacetate by pyruvate carboxylase) [27]. The overall net flux toward the TCA cycle generated by the concerted action of the carboxylating and decarboxylating enzymes was rather low (14.8%). Therefore, this anaplerotic route alone was not sufficient to replenish the TCA cycle, which was continuously depleted by the anabolic requirement for its intermediates oxaloacetate (17%) and 2-oxoglutarate (10%). Note that the cells recruited the glyoxylate shunt as an anaplerotic pathway. At the level of isocitrate, approximately 20% of carbon was channeled into the shunt, matched by a relative flux of 12%. The activity of this pathway seemed surprising at first, because it is typically not required in glucose-grown cells. We therefore performed *in-vitro* measurements to detect isocitrate lyase, the key enzyme of this pathway. Indeed, isocitrate lyase was present in the cytoplasm (124±1 mU· (mg protein)^−1^). Further studies showed that acetate as sole carbon source activated isocitrate lyase by approximately a factor of four (Figure S4).

### Metabolic analysis of clinical isolates using [1-^13^C] glucose

**Figure 3:**
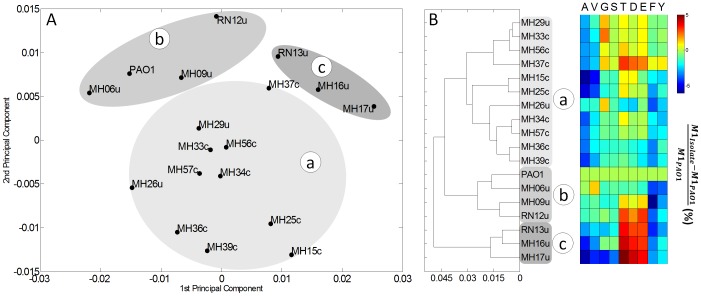
Statistical analysis of carbon core metabolism of uropathogenic *P. aeruginosa* isolates and *P. aeruginosa* PAO1 based on ^13^C-labeled amino acid enrichment data from the tracer studies. Principal component analysis provided a clustering of the strains according to the two major components (A). Hierarchical cluster analysis revealed the degree of similarity, shown as a Euclidian tree (B). The relative fraction of the single labeled (M1) mass isotopomer of each amino acid was considered after normalization to the value of *P. aeruginosa* PAO1. The relative ^13^C-enrichment is displayed in color. The amino acids are denoted by their single letter code.

The *P. aeruginosa* isolates grown on [1-^13^C] glucose exhibited balanced growth and reached a metabolic steady state (Figure S5). The ^13^C-labeling patterns of proteinogenic amino acids (Table S2) were first explored by unsupervised statistical analysis because they were informative for directly discriminating between different types of metabolism and pathway use [38] and provided an initial qualitative overview of the strains. Briefly, the ^13^C-data were analyzed using PCA (Figure 3A) and HCA (Figure 3B). The 18 strains clustered into three subgroups, designated as clusters *a*, *b* and *c* in the Euclidian tree. This revealed site-specific metabolism. Clusters *b* and *c* comprised only isolates from urinary tract infections. In contrast, all strains from catheter-related infections grouped in cluster *a*. The only strains assigned to cluster *a* from the urinary tract infections were MH26u and MH29u. The clustering resulted from differences in distinct amino acids and were therefore of metabolic origin. A prominent example is the high enrichment of label in threonine, aspartate, and glutamate, which was specific for the strains of cluster *c* (Figure 3B). The three clusters were also identified using PCA (Figure 3A), which described 98% of the labeling information.

### Metabolic flux of *P. aeruginosa* isolates

**Figure 4:**
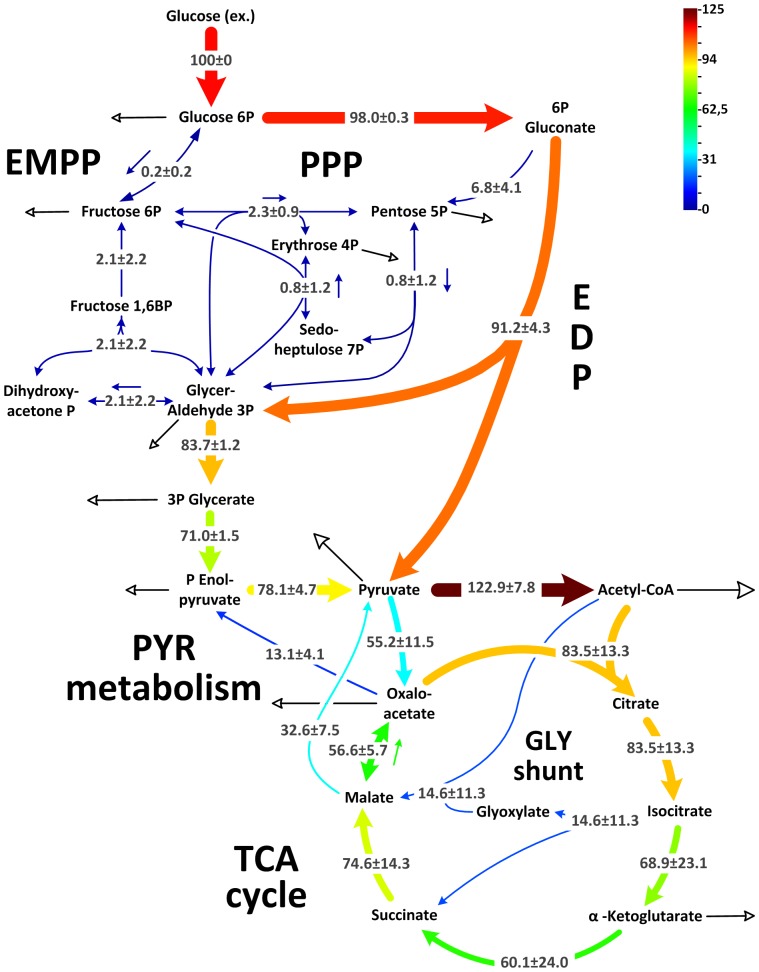
In vivo carbon flux distributions in central metabolism of uropathogenic *P. aeruginosa* isolates during growth on glucose. Flux is given as average flux of all strains and is expressed as a molar percentage of the average glucose uptake rate of all strains (8.6 mmol g^−1^ h^−1^, calculated from the individual rates in Table 1). Open arrows indicate flux toward biomass. For reversible reactions, the direction of net flux is indicated by a dashed arrow. The errors given for each flux reflect the corresponding 90% confidence intervals. The full flux data sets are presented in Supporting Information. Metabolic and isotopic steady states are ensured by constant stoichiometry, kinetics, and the constant ^13^C-labeling patterns of recruited metabolites during cultivation (see Figure S1, Figure S5). Abbreviations are as follows: Entner-Doudoroff pathway (EDP), Embden-Meyerhof-Parnas pathway (EMPP), pentose phosphate pathway (PPP), glyoxylate (Gly) shunt, and tricarboxylic acid (TCA) cycle.

**Figure 5:**
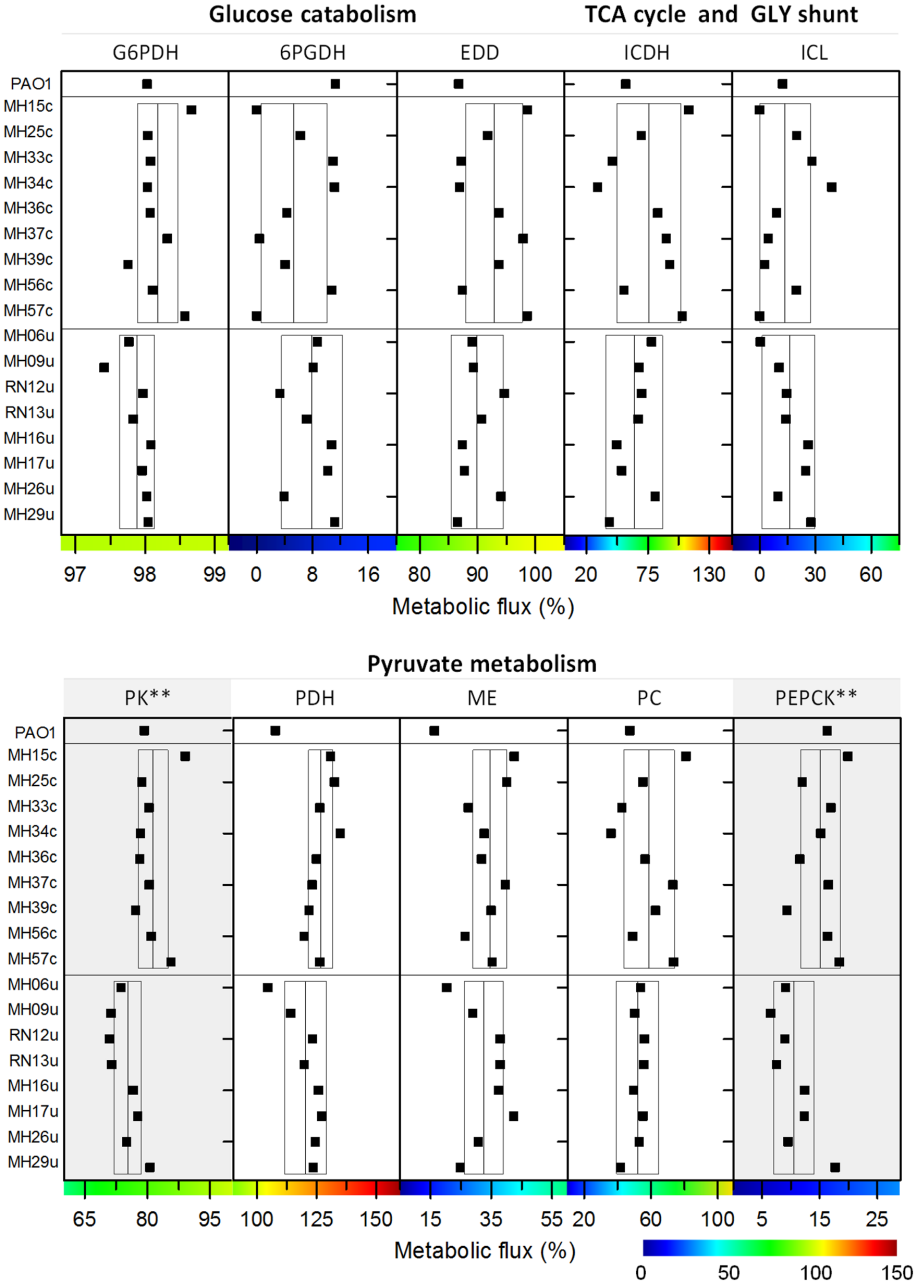
In vivo carbon fluxes through central metabolic pathways of uropathogenic *P. aeruginosa* during growth on glucose. The data reflect the individual flux for each isolate. All fluxes are expressed as a molar percentage of the corresponding specific glucose uptake rate (Table 1). Statistical differences of flux between strains from urinary tract infections and from catheter-associated infections were assessed using a *t* test. Significant differences (**P<0.05) are indicated. Abbreviations are as follows: glucose 6-phosphate dehydrogenase (G6PDH), 6-phosphogluconate dehydrogenase (6PGDH), 6-phosphogluconate dehydratase (EDD), isocitrate dehydrogenase (ICDH), isocitrate lyase (ICL), pyruvate kinase (PK), pyruvate dehydrogenase (PDH), malic enzyme (ME), pyruvate carboxylase (PC), and phosphoenolpyruvate carboxykinase (PEPCK). The full flux data sets for all strains are presented in the Supporting Information.

The full set of flux analysis as well as all experimental and simulated labeling patterns, which reflect optimal fit, are presented in Table S2. Figure 4 provides an integrated view of pathway use. Briefly, flux through each reaction displays the average value of all strains, whereas the deviation indicates the corresponding variability. Flux through the initial metabolic pathways, i.e., EDP, PPP and EMPP, respectively, was conserved. This was inferred from the flux data of the single isolates (Figure 5) where the corresponding pathways showed similar activity.

Only three catheter-associated strains (MH15c, MH37c, and MH57c) did not use 6-phosphogluconate dehydrogenase, whereas all other strains channeled carbon through this enzyme into the PPP. Using an *in-vitro* assay, phosphofructokinase activity was not detected in any isolate (<0.01 mU (mg protein)^−1^). All isolates, as well as PAO1, recruited pyruvate carboxylase but not PEP carboxylase (Figure 2). Interestingly, flux varied downstream from the pyruvate node. This involved the TCA cycle and the reactions interconnecting the cycle with the glycolytic intermediates, i.e., the anaplerotic and gluconeogenetic reactions.

The most variable metabolic reactions were localized around the isocitrate node, where the isolates differed significantly in the flux partitioning between the glyoxylate shunt and the TCA cycle (Figure 4). This is even more apparent when inspecting individual strains (Figure 5). In certain isolates, the glyoxylate shunt was completely inactive, whereas others showed high flux (44%). As exemplified by MH34c, strains with an active shunt showed isocitrate lyase activity (see Figure S4). Similarly, the TCA cycle (17–112%) and other enzymes, positioned near the pyruvate node varied strongly in flux. Note that flux through pyruvate kinase and phosphoenolpyruvate carboxykinase was significantly different between the two clinical subgroups (Figure 5). The catheter-associated isolates carried higher flux through these reactions (*P*<0.05) compared with strains isolated from patients with urinary tract infections.

## Discussion

The present study describes the analysis of metabolic fluxes in *P. aeruginosa* PAO1 as well as uropathogenic isolates and provides novel insights into function and regulation of carbon core metabolism of this important pathogen. We show here that *P. aeruginosa* isolates catabolize glucose through the EDP with fully respiratory metabolism and without overflow (Figure 2, Figure 4, Figure 5). They further recruit the oxidative and non-oxidative PPP exclusively for biosynthesis, but do not exhibit a functional EMPP. All strains utilize pyruvate carboxylase but not PEP carboxylase (Figure 2), and the glyoxylate shunt operates as anaplerotic pathway. Hierarchical clustering of the strains according to their flux reveals a site-specific metabolism among the isolates, which might indicate that *P. aeruginosa* differently adapts to its environment: the urinary tract and the surface of catheters infections, respectively. The isolates differ strongly in glucose uptake rate and growth efficiency. Faster uptake of nutrients and their more efficient conversion into biomass might provide an advantage to compete and persist during infection. However, one should be cautious with this particular interpretation due to potential metabolic differences, caused by different levels of oxygen in our aerobic experimental setup and the oxygen limited infection environments, in which *P. aeruginosa* thrives [39].

### The exclusive use of the EDP as a glycolytic strategy is conserved among *P. aeruginosa* and other pseudomonads

The predominant use of the EDP is identical to that of other glucose-grown pseudomonads, including *P. putida*, *P. fluorescens*, and *P. denitrificans* previously studied at the flux level (Table 2). This finding is attributed to the lack of phosphofructokinase, an essential enzyme of the alternative glycolytic EMPP [27]. However, this glycolytic strategy is uncommon among prokaryotes, and only 12% of bacteria rely solely on the EDP [40], whereas the more energy-efficient EMPP is nearly ubiquitous [41]. However, the EDP mediates superior resistance to oxidative stress [42]. In this study, the expression of a functional EMPP in *P. putida*, which forced the organism to redirect flux from the natural ED route, significantly decreased its robust response to oxidative stress [42], because the EMP pathway does not yield NADPH required by various antioxidant defense mechanisms [43]–[46]. In contrast, the ED pathway provides NADPH [40].

**Table 2:** Metabolic flux in *P. aeruginosa* PAO1 and select gram-negative and gram-positive bacteria.

Organism	EDP [%]	EMPP [%]	PPP [%]	Gly shunt [%]	Reference
*P. aeruginosa* PAO1	87	-*	11	12	this work
*P. putida* KT2440	89	-	11	n.d.**	[27]
*P. fluorescens*	91	-	9	n.d.	[65]
*E. coli*	3	73	25	0	[53]
*C. glutamicum*	-	67	33	0	[66]

Flux refers to the Entner-Doudoroff pathway (EDP), Embden-Meyerhof-Parnas pathway (EMPP), pentose phosphate pathway (PPP), and glyoxylate (Gly) shunt and reflects relative values normalized to the corresponding glucose uptake rate, defined as 100%.

* functional pathway not encoded.

** n.d. = not determined.

Accordingly, bacteria that reside in the environment, such as *P. putida*, favor the ED pathway to better cope with naturally occurring oxidative stress [42]. We conclude, therefore, that the high ED flux of *P. aeruginosa* provides a major benefit for this pathogen, which must counteract oxidative stress imposed by the host as a response to infection. Moreover, *P. aeruginosa* senses the redox state and switches its metabolism as a reaction to the host immune defense and other oxidative stress conditions, which involves the activation of catalase, thiol reductase, and superoxide dismutase, all of which use NADPH as a cofactor [47].

### Anaplerotic and TCA cycle flux of uropathogenic *P. aeruginosa* reveal high plasticity

Microevolution of *P. aeruginosa* during chronic pulmonary infection creates a metabolic shift to support adaptation to the host environment [7], [48]. Transcriptome profiling has shown that this predominantly involves its carbon core machinery [11]. Briefly, adaptive evolution affected only specific pathways such as pyruvate metabolism, the TCA cycle, and the linked pathways of anaplerosis and gluconeogenesis. In contrast, the reactions that metabolize glucose to pyruvate via the EDP remained unchanged.

**Figure 6:**
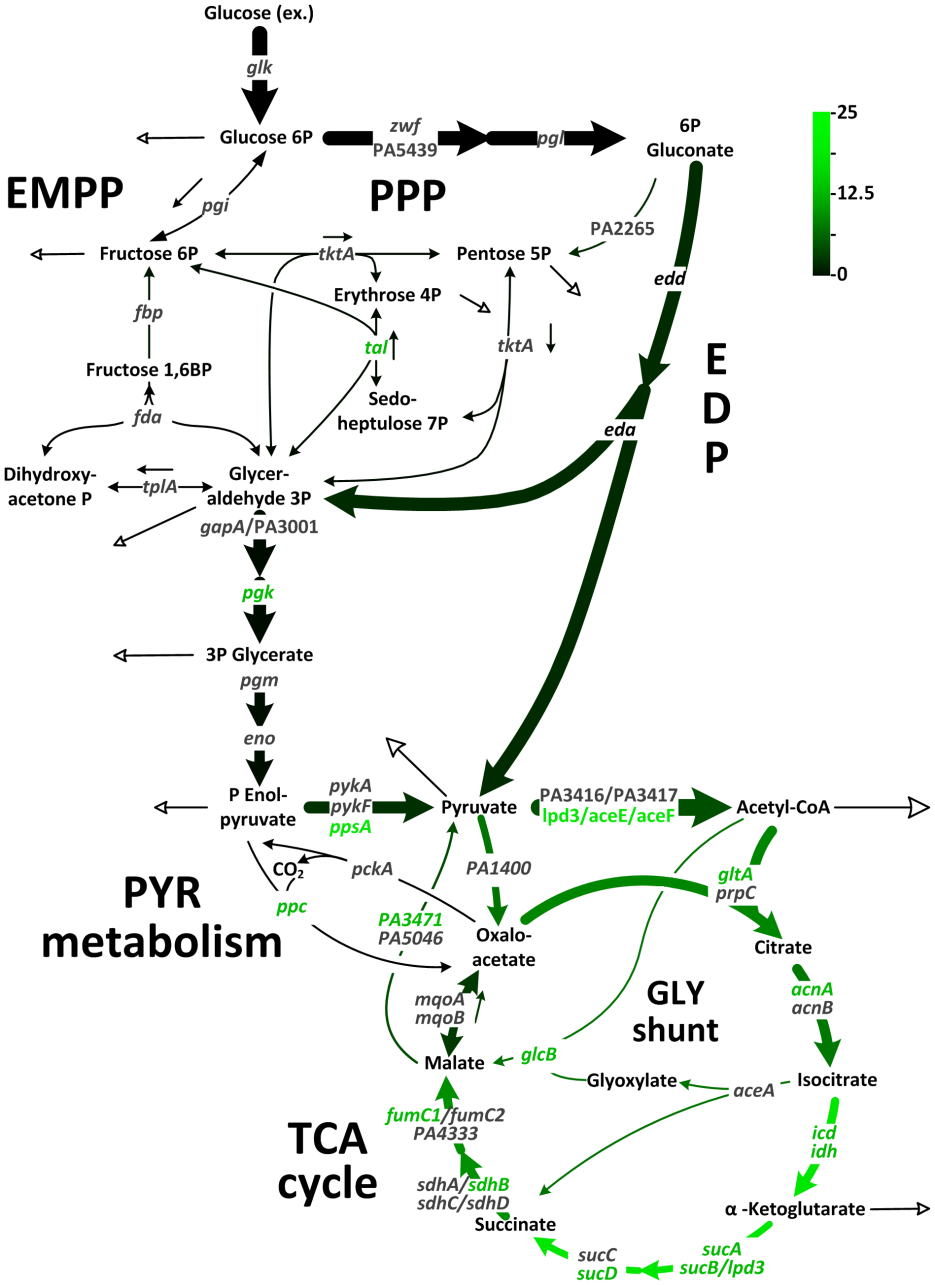
Metabolic adaption of pathogenic *P. aeruginosa*. The network representation integrates changes of flux determined for uropathogenic isolates and *P. aeruginosa* PAO1 (this work) and changes in transcription transcript level previously determined for successive isolates of *P. aeruginosa* from patients with chronic cystic fibrosis [11]. The variation of metabolic flux among strains is indicated by color (black = conserved, green = changed). Genes with changed or unchanged transcript levels are shown in green or black, respectively. Abbreviations are as follows: Entner-Doudoroff pathway (EDP), Embden-Meyerhof-Parnas pathway (EMPP), pentose phosphate pathway (PPP), glyoxylate (Gly) shunt, and tricarboxylic acid (TCA) cycle.

**Figure 7:**
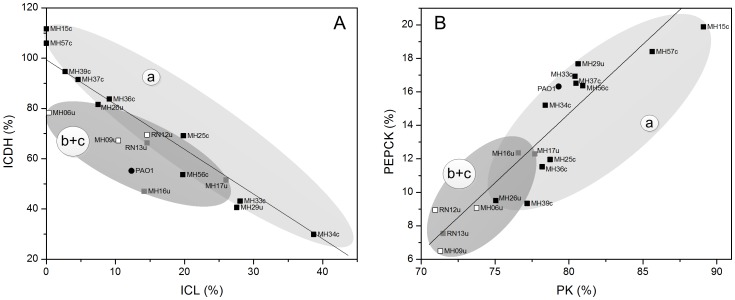
Niche-specific metabolic flux in uropathogenic *P. aeruginosa*, including flux through isocitrate dehydrogenase and isocitrate lyase (A) and through pyruvate kinase and phosphoenolpyruvate carboxykinase (B). Each data point refers to a distinct isolate. The clustering represents statistical analysis using principal components (see Figure 4) that discriminated between isolates from catheter-associated infections (cluster *a*) and strains from urinary tract infections (clusters *b* and *c*). The full flux data sets for all strains are presented in Supporting Information. All flux values are normalized to the average glucose uptake rate for all strains (100%).

The integration of observations of distinct metabolic changes in *P. aeruginosa* during chronic lung infection determined at the transcript level [11] taken together with the metabolic flux changes in uropathogenic isolates presented here, reveal a striking picture (Figure 6). It seems that the pathogen undergoes a similar metabolic adaptation in two very different infection environments: the lung and the urinary tract. The conserved upstream steps of metabolism, i.e. conversion of glucose to pyruvate, are combined with variations in metabolic flux through the downstream steps of metabolic pathways among all strains. For example, the isolates varied in the flux-partitioning ratio at isocitrate, i.e., toward the glyoxylate shunt and TCA cycle (Figure 7 A). Further, flux at the PEP pool showed significant differences (Figure 7B). Similarly, these reactions were also affected during chronic lung infection [11] (Figure 6). This may reflect a more general underlying strategy that allows *P. aeruginosa* to adapt to the host environment. The validity of this conclusion is limited by our study's use of isolates from different patients, which did not allow a time-resolved reconstruction of the evolving flux changes during infection. Nevertheless, the alterations in metabolism detected among these isolates are likely involved in adaptation and supports their survival in the host. Further flux studies on strains, experimentally evolved in the laboratory [49], [50], and on evolutive lineages of clinical isolates [10], [11] seem straightforward to confirm this assumption.

### The isocitrate node displays a flexible switch-point in the metabolism of *P. aeruginosa*

The glyoxylate shunt, comprising isocitrate lyase and malate synthase, is widespread [51]. First discovered in *P. aeruginosa* [52], it has been commonly considered as a by-pass of the TCA cycle to assimilate carbon from C2 compounds [22] and to overcome limitations in the junction between glycolysis and TCA cycle under stress [53]. The use of the shunt in non-pathogenic bacteria grown on glucose was not significant (Table 2). In contrast, most of the *P. aeruginosa* clinical isolates exhibited significant flux through this pathway (Figure 5). The presence of the glyoxylate shunt, a bit surprising at first, was confirmed by enzyme assays (Figure S4). Its use in the presence of glucose differed from other bacteria, which repress the shunt (Figure S4), as shown previously [54].

The enhancement of isocitrate lyase activity by a factor of 4 for acetate-grown *P. aeruginosa* indicates transcriptional regulation of the pathway, which is known for *C. glutamicum* [54]. At the level of isocitrate dehydrogenase, the glyoxylate shunt competes with the TCA cycle, i.e., cells can direct carbon toward either pathway. Consistent with this, flux through both pathways is coupled (Figure 7A). Here, the isolates differed significantly in the relative activation of the glyoxylate shunt. Note that the shunt plays a major role in bacterial pathogenesis [55]. For example, isocitrate lyase is up-regulated in *P. aeruginosa* during infection [56]. Mutants lacking a functional glyoxylate pathway show reduced virulence in plants and mammals [57]. However, the shunt is essential for the survival of other pathogens inside the host, including *Mycobacterium tuberculosis* [58], *Salmonella enterica* [59], and *Rhodococcus equi* [60]. *P. aeruginosa* isolates show an enhanced turnover of fatty acids and lipids that are abundantly found in the host's environment [11]. The metabolism of such substrates ultimately requires the glyoxylate shunt to metabolize the C2 intermediate acetyl CoA formed during their degradation. Therefore, the changed flux might reflect an adaptation to nutrients available in the host.

### Uropathogenic *P. aeruginosa* is optimized for growth efficiency

**Figure 8:**
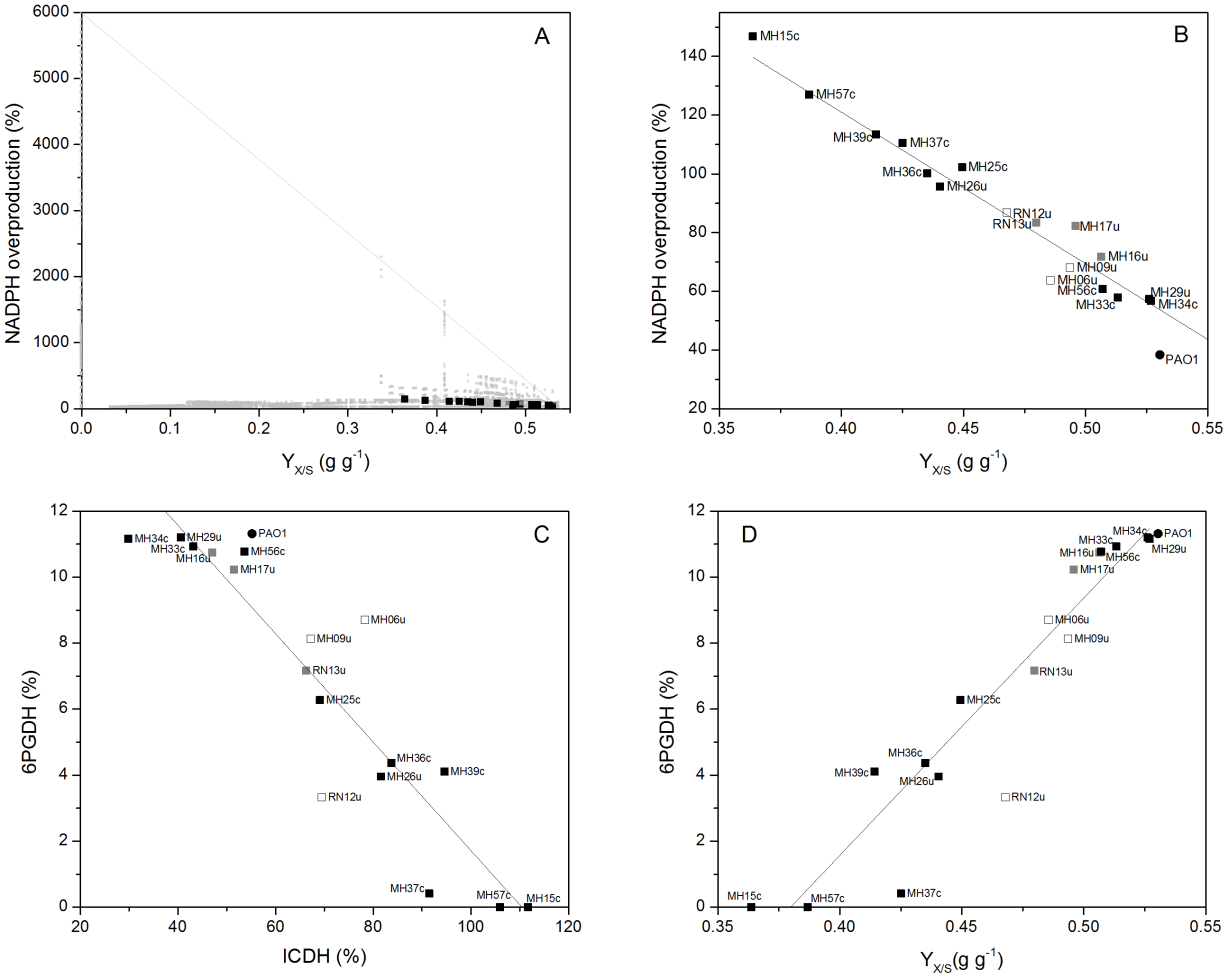
Metabolic properties of uropathogenic *P. aeruginosa* isolates and *P. aeruginosa* PAO1. Integration of flux phenotypes into the theoretical flux space on basis of anabolism (considering the biomass yield coefficient Y_x/s_, given in Table 1) and of NADPH metabolism (considering the NADPH balance as described below). In order to evaluate individual flux phenotypes, experimental values for biomass yield and apparent NADPH excess, respectively, (black squares) are integrated into the flux space created by elementary flux mode analysis (grey squares). Most strains localize close to the growth optimum. (A), Correlation of biomass formation and NADPH metabolism (B), contribution of isocitrate dehydrogenase and 6-phosphogluconate dehydrogenase to supply of reducing power (C), correlation of biomass formation and flux through the oxidative pentose phosphate pathway (D). NADPH metabolism was inspected by balancing of the redox cofactor. For this purpose, the NADPH formation flux by concerted action of glucose 6-phosphate dehydrogenase, 6-phosphogluconate dehydrogenase, isocitrate dehydrogenase, and malic enzyme was balanced with the NADPH consumption flux for anabolism. For all strains, NADPH formation was higher than consumption. The resulting apparent excess flux of NADPH supply is presented here. The full flux data sets for all strains are presented in Supporting Information. The flux values are normalized to the corresponding glucose uptake rate for each strain (set to 100%).

For the metabolic network of *P. aeruginosa*, computer-based analysis yielded 12,955 elementary flux modes, each a unique, minimal combination of reactions with fluxes that support steady state operation of cellular metabolism [34]. Together, all modes spanned the theoretical flux space [35]. Here, we focused on growth efficiency and redox metabolism of *P. aeruginosa*. The model allowed for NADPH overproduction. Particularly, we were interested in scenarios, which produce more NADPH than needed for anabolism, i.e. reveal apparent NADPH excess. We assumed that this extra amount of reducing power approximately reflects tolerance against oxidative stress, because NADPH drives many antioxidant mechanisms [43]–[45].Two-dimensional visualization grouped the obtained elementary modes according to their efficiency for growth and NADPH production (Figure 8 A). Therefore, we defined the yield coefficient for biomass (Y_X/S_) associated with a particular mode as measure of growth efficiency. As measure of NADPH metabolism, we defined the surplus NADPH that resulted from balancing of this cofactor: For each mode, the NADPH formation flux through glucose 6-phosphate dehydrogenase, 6-phosphogluconate dehydrogenase, isocitrate dehydrogenase, and malic enzyme was balanced with the NADPH consumption flux for anabolism.

In order to evaluate the isolates, we placed their flux phenotypes, i.e. the experimental biomass yield coefficient (Figure 8 A) and the flux of apparent NADPH overproduction (Figure 8 B) into this space of feasible physiological states and looked for their distance to certain points of optimality.

The elementary modes (grey squares) span a triangle with the extremes at the corners, reflecting the corresponding theoretical maximum yield for biomass (Y_X/S_ = 0.54 g·g^−1^) and the maximum flux for NADPH overproduction (6,000%), respectively. The experimentally determined yields and the NADPH overproduction flux of all isolates (black squares) clearly localize close to optimum growth (Figure 8 A). We conclude that the metabolism of these strains is optimized for growth. However, the metabolic network exhibits a significant potential for enhancing NADPH overproduction, if needed. Growth efficiency occurred at the expense of the NADPH supply, because flux closely coupled in the isolates (Figure 8 B). Therefore, the amount of extra NADPH, not needed for anabolism, was decreased in efficient growers. However, they all exhibited an apparent excess, which was not required for anabolism.

We conclude that, under the conditions studied, *P. aeruginosa* recruits a transhydrogenase to direct the extra NADPH to NADH and the respiratory chain for energy generation. Under stress, the extra amount of NADPH, which is not required for anabolism, could immediately serve for protection against oxidative stress [46].Two nucleotide transhydrogenases, the soluble Sth and the membrane-bound Pnt, respectively, are annotated in the genome of *P. aeruginosa* [24] and might be involved in this inter conversion process. It is unlikely that the transhydrogenases operate in the NADH-to-NADPH direction, although the flux data do not allow a definite conclusion.

### Improved growth efficiency relates to preferential use of the PPP but reduced the production of excess NADPH

The *P. aeruginosa* isolates differed in their relative use of the oxidative PPP (Figures 8 C and D), and this is directly related to their growth efficiency. Strains with an increased biomass yield recruited the PPP to a higher extent. We conclude that the activated PPP represented by its entry enzyme, 6-phosphogluconate dehydrogenase, served for biosynthesis. First, it supplied anabolic precursors such as erythrose 4-phosphate and ribose 5-phosphate. Second, it compensated for reduced flux through isocitrate dehydrogenase that catalyzes the synthesis of NADPH in the TCA cycle, which was reduced as a function of increased biomass formation. Thus, the PPP may provide a significant source of redox potential. Similarly, these enzymes complement each other, supplying NADPH as in other bacteria [32].

### *P. aeruginosa* shows niche-specific traits in carbon core metabolism related to the type of infection

The flux data indicate that the metabolism of strains isolated from urinary tract infections differed from those isolated from catheter-associated infections. This represents potential adaptations to the specific conditions during infection. The strains were grouped into three clusters according to flux level. In particular, the differences involved the ^13^C-labeling of amino acids generated by the TCA cycle (Figure 3 B) and the flux at the junction between the cycles, the pyruvate node, and the glyoxylate shunt (Figures 5, 6, and 7A and B). Other studies demonstrate site-specific phenotypes of *P. aeruginosa* isolates [16], [61], [62]. These differences include extracellular enzymes such as phospholipase [16] or elastase [63], reflecting specific adaptation to nutrient status in the corresponding host environment. Changes in biofilm formation, cell adhesion, and polymer formation indicate adaptions to sessile life styles [16]. Note that the clustering of the *P. aeruginosa* isolates at the phenotypic flux level (Figure 3 B) was completely different from a previously reported clustering at the genetic level [16]. At seems clear that a different number of changes in the genome could have the same effect on cell activity or even no effect [64]. Consequently, characterization of function, i.e., metabolic level, promises to provide a more direct understanding of the adaption processes. In particular, central metabolic pathways, activated during the infection, appear relevant. Future metabolic flux analysis of direct evolutionary lineages of *P. aeruginosa* [10], [11] and under conditions of oxygen limitation, typically present in the afflicted tissues in which these isolates thrive [39], should be straightforward and promises to shed more light on this interesting question.

## Supporting Information

Figure S1 **Growth characteristics and validation of the experimental approach exemplified for the reference strain** ***P. aeruginosa*** **PAO1.** Cultivation profile on minimal glucose medium (A). Metabolic and isotopic steady-state are visualized by constant yield for biomass, derived as slope from the profiles of consumed glucose concentration and formed cell dry weight (B). Isotopic steady state is indicated by constant labeling patterns over time, as shown for single-labeled mass isotopomers (M1) of [M-57] amino acid fragments (C). The growth behavior in shake flasks and deep-well plates was identical regarding growth stoichiometry (B) and ^13^C labeling data (D) justifying the joint use of data. Accordingly, cell dry weight measurements from shake flask cultures could be integrated with growth and labeling data from deep-well plates for flux calculations, when needed. Experiments were performed in three replicates.
(PDF)

Figure S2 **Dissolved oxygen during cultivation of** ***P. aeruginosa*** **on minimal medium in shake flask culture.** To ensure sufficient aeration during cultivation, the level of dissolved oxygen was monitored on-line. As exemplified for *P. aeruginosa* PAO1, the oxygen level was above 80% of saturation so that fully aerobic conditions were given (one of three replicates shown). The excellent agreement of growth kinetics and stoichiometry and of the ^13^C labeling fingerprint (Figure S1), confirmed that this was obviously also the case for the deep-well plate cultures.
(PDF)

Figure S3 **Growth characteristics of uropathogenic** ***P. aeruginosa*** **isolates.** Cultivation profiles on minimal glucose medium (three biological replicates each). For none of the strains, extracellular by-products were detected.
(PDF)

Figure S4 **Enzymatic analysis of isocitrate lyase as key enzyme of the glyoxylate shunt.** ***C. glutamicum*** and *P. aeruginosa* were cultivated in minimal medium, supplemented either with 40 mM acetate or with 14 mM glucose as sole carbon source, respectively.
(PDF)

Figure S5 **Metabolic profiles of consumed glucose concentration and formed cell dry weight of clinical** ***P. aeruginosa*** **isolates on minimal glucose medium (three biological replicates each).** Metabolic steady-state is inferred from the constant yield for biomass, derived as slope from the profiles.
(PDF)

Table S1 Refinement of the cellular composition of *P. aeruginosa* by quantification of the alginate capsule and the corresponding anabolic demand for F6P for its biosynthesis. For quantification, alginate was detached from the cells, and then analyzed by the sulfamate-biphenyl method [1]. Shortly, cells were harvested, washed once with deionized water. Subsequently, the alginate was detached by shaking for 5 h (300 µL 0.14 M NaCl suspension, 1400 min^−1^, 25°C). The supernatant (200 µL), obtained by centrifugation (15 min, 16,000× *g*, 4°C), was then amended with 20 µL 4 M sulfamate and with 1.2 mL 0.075 M tetraborate, dissolved in concentrated H_2_SO_4_, and then incubated for 20 min at 99°C. Afterwards, the suspension was transferred to an ice bath (5 min), followed by addition of 40 µL 0.15% 3-hydroxybiphenyl, dissolved in 0.5% NaOH. The mixture was incubated for 10 min at room temperature. Alginate was quantified by photometry at 525 nm, using isolated alginate from mucoid *P. aeruginosa* FRD1 as external standard [2]. The data reflect the F6P demand for alginate biosynthesis and are given as relative flux, normalized to the specific glucose uptake rate (Table 1). These values were additionally considered in the anabolic requirement for flux analysis by correcting the demand for F6P (Table S2). Generally, the requirement was low.
(PDF)

Table S2 Supporting information on metabolic flux analysis. The data include the ^13^C labelling analysis of proteinogenic amino acids by GC-MS for all strains, the entire set of estimated metabolic fluxes, and information on the goodness of fit, i.e. the comparison of experimental and simulated labelling data, corresponding to the optimized fit.
(XLSX)
