# Supplementary material for: Robustness and Plasticity of Metabolic Pathway Flux among Uropathogenic Isolates of Pseudomonas aeruginosa
Source: PLoS One. 2014 Apr 7;9(4):e88368. doi: 10.1371/journal.pone.0088368 (PMC3977821; doi:10.1371/journal.pone.0088368)
Supplement: Figure S1 — Growth characteristics and validation of the experimental approach exemplified for the reference strain P. aeruginosa PAO1. Cultivation profile on minimal glucose medium (A). Metabolic and isotopic steady-state are visualized by constant yield for biomass, derived as slope from the profiles of consumed glucose concentration and formed cell dry weight (B). Isotopic steady state is indicated by constant labeling patterns over time, as shown for single-labeled mass isotopomers (M1) of [M-57] amino acid fragments (C). The growth behavior in shake flasks and deep-well plates was identical regarding growth stoichiometry (B) and 13C labeling data (D) justifying the joint use of data. Accordingly, cell dry weight measurements from shake flask cultures could be integrated with growth and labeling data from deep-well plates for flux calculations, when needed. Experiments were performed in three replicates. (PDF) [file pone.0088368.s001.pdf]

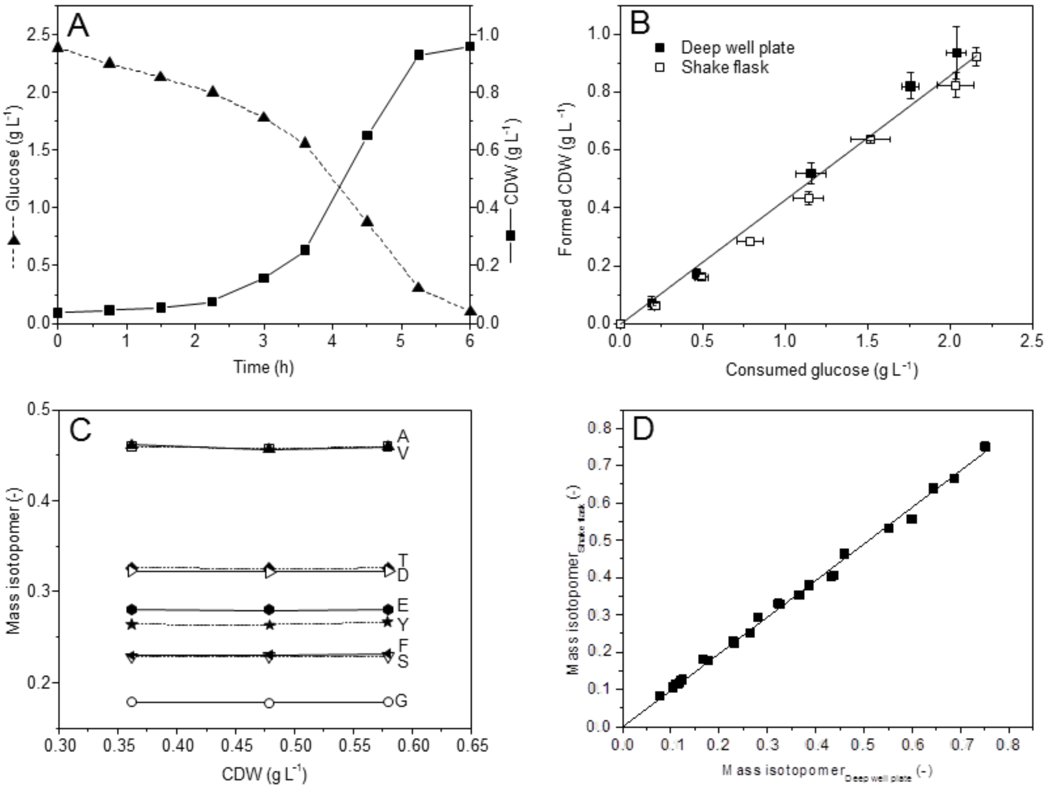

Figure S1. Growth characteristics and validation of the experimental approach exemplified for the reference strain *P. aeruginosa* PAO1. Cultivation profile on minimal glucose medium (A). Metabolic and isotopic steady-state are visualized by constant yield for biomass, derived as slope from the profiles of consumed glucose concentration and formed cell dry weight (B). Isotopic steady state is indicated by constant labeling patterns over time, as shown for single-labeled mass isotopomers (M1) of [M-57] amino acid fragments (C). The growth behavior in shake flasks and deep-well plates was identical regarding growth stoichiometry (B) and ^{13}C labeling data (D) justifying the joint use of data. Accordingly, cell dry weight measurements from shake flask cultures could be integrated with growth and labeling data from deep-well plates for flux calculations, when needed. Experiments were performed in three replicates.
