# Supplementary material for: Robustness and Plasticity of Metabolic Pathway Flux among Uropathogenic Isolates of Pseudomonas aeruginosa
Source: PLoS One. 2014 Apr 7;9(4):e88368. doi: 10.1371/journal.pone.0088368 (PMC3977821; doi:10.1371/journal.pone.0088368)
Supplement: Figure S2 — Dissolved oxygen during cultivation of P. aeruginosa on minimal medium in shake flask culture. To ensure sufficient aeration during cultivation, the level of dissolved oxygen was monitored on-line. As exemplified for P. aeruginosa PAO1, the oxygen level was above 80% of saturation so that fully aerobic conditions were given (one of three replicates shown). The excellent agreement of growth kinetics and stoichiometry and of the 13C labeling fingerprint (Figure S1), confirmed that this was obviously also the case for the deep-well plate cultures. (PDF) [file pone.0088368.s002.pdf]

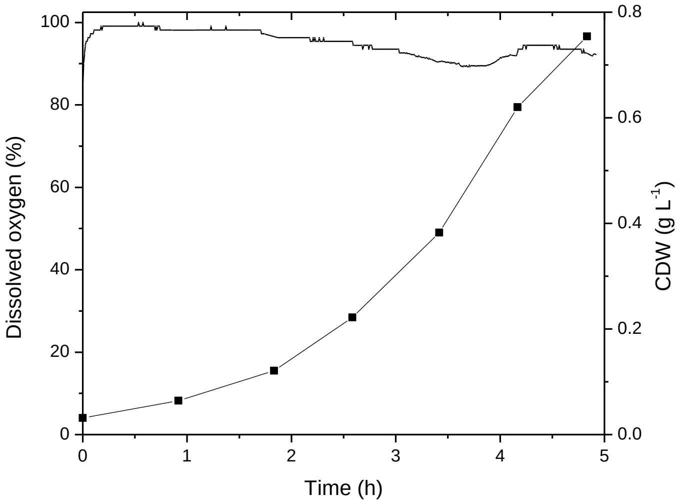

Figure S2. Dissolved oxygen during cultivation of *P. aeruginosa* on minimal medium in shake flask culture. To ensure sufficient aeration during cultivation, the level of dissolved oxygen was monitored on-line. As exemplified for *P. aeruginosa* PAO1, the oxygen level was above 80 % of saturation so that fully aerobic conditions were given (one of three replicates shown). The excellent agreement of growth kinetics and stoichiometry and of the ^{13}C labeling fingerprint (Figure S1), confirmed that this was obviously also the case for the deep-well plate cultures.
