# Supplementary material for: Robustness and Plasticity of Metabolic Pathway Flux among Uropathogenic Isolates of Pseudomonas aeruginosa
Source: PLoS One. 2014 Apr 7;9(4):e88368. doi: 10.1371/journal.pone.0088368 (PMC3977821; doi:10.1371/journal.pone.0088368)
Supplement: Figure S3 — Growth characteristics of uropathogenic P. aeruginosa isolates. Cultivation profiles on minimal glucose medium (three biological replicates each). For none of the strains, extracellular by-products were detected. (PDF) [file pone.0088368.s003.pdf]

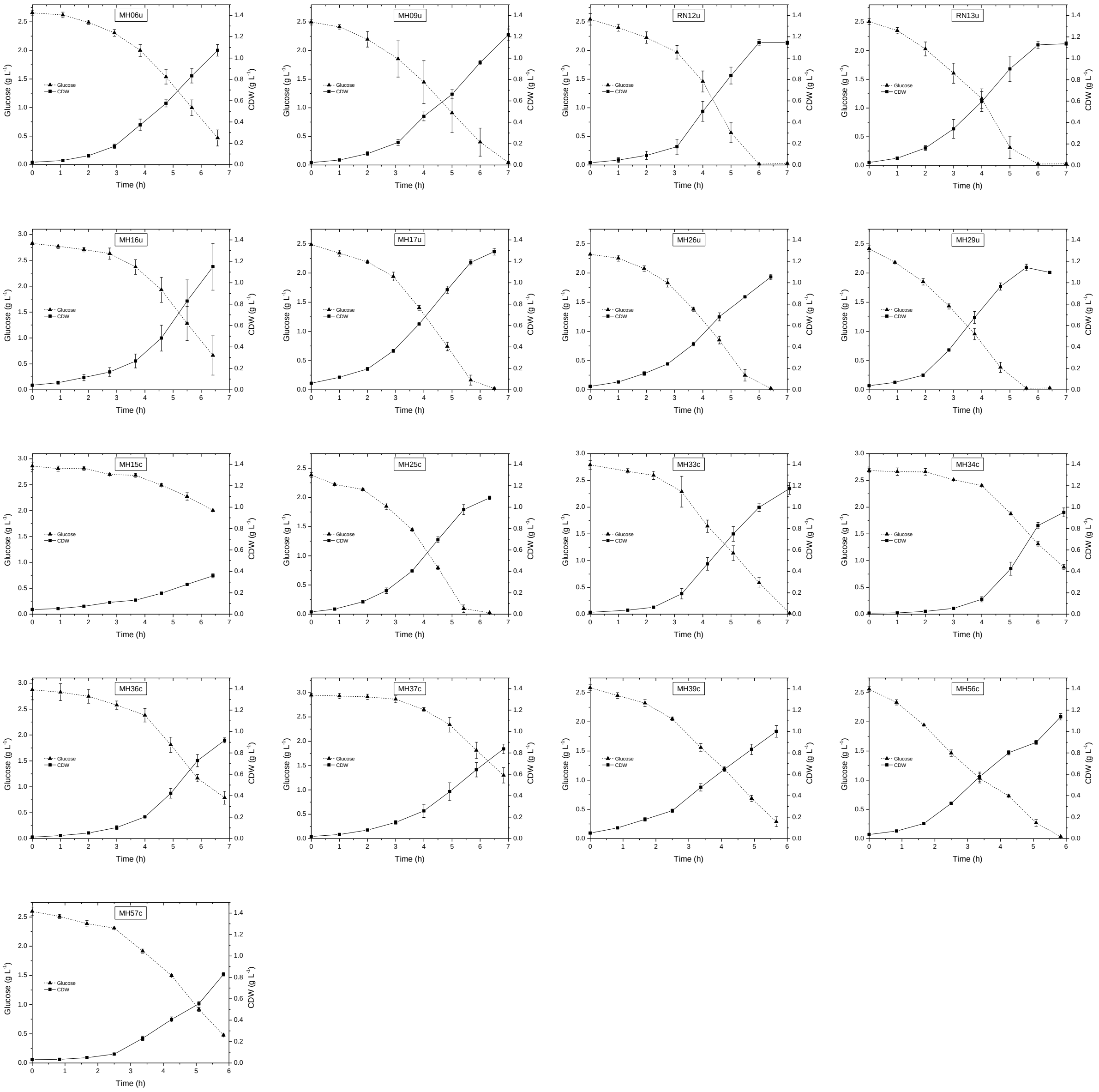

Figure S3. Growth characteristics of uropathogenic *P. aeruginosa* isolates. Cultivation profiles on minimal glucose medium (three biological replicates each). For none of the strains, extracellular by-products were detected.
