# Supplementary material for: Robustness and Plasticity of Metabolic Pathway Flux among Uropathogenic Isolates of Pseudomonas aeruginosa
Source: PLoS One. 2014 Apr 7;9(4):e88368. doi: 10.1371/journal.pone.0088368 (PMC3977821; doi:10.1371/journal.pone.0088368)
Supplement: Figure S4 — Enzymatic analysis of isocitrate lyase as key enzyme of the glyoxylate shunt. C. glutamicum and P. aeruginosa were cultivated in minimal medium, supplemented either with 40 mM acetate or with 14 mM glucose as sole carbon source, respectively. (PDF) [file pone.0088368.s004.pdf]

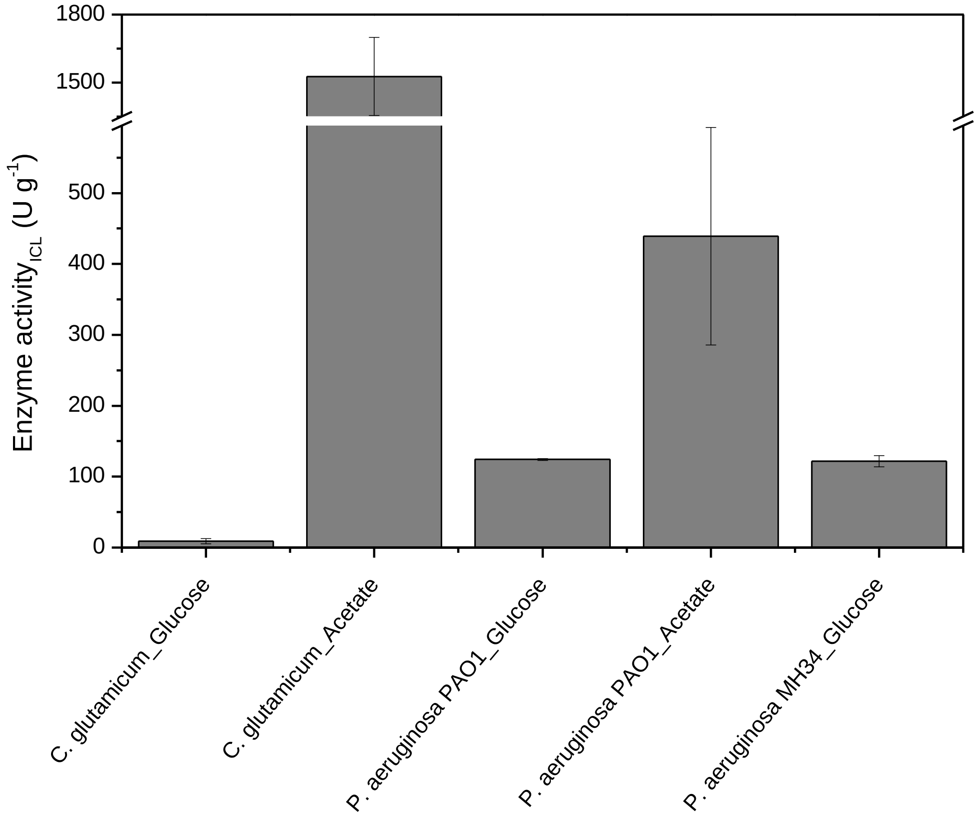

Figure S4. Enzymatic analysis of isocitrate lyase as key enzyme of the glyoxylate shunt. *C. glutamicum* and *P. aeruginosa* were cultivated in minimal medium, supplemented either with 40 mM acetate or with 14 mM glucose as sole carbon source, respectively.
