# Supplementary material for: Robustness and Plasticity of Metabolic Pathway Flux among Uropathogenic Isolates of Pseudomonas aeruginosa
Source: PLoS One. 2014 Apr 7;9(4):e88368. doi: 10.1371/journal.pone.0088368 (PMC3977821; doi:10.1371/journal.pone.0088368)
Supplement: Figure S5 — Metabolic profiles of consumed glucose concentration and formed cell dry weight of clinical P. aeruginosa isolates on minimal glucose medium (three biological replicates each). Metabolic steady-state is inferred from the constant yield for biomass, derived as slope from the profiles. (PDF) [file pone.0088368.s005.pdf]

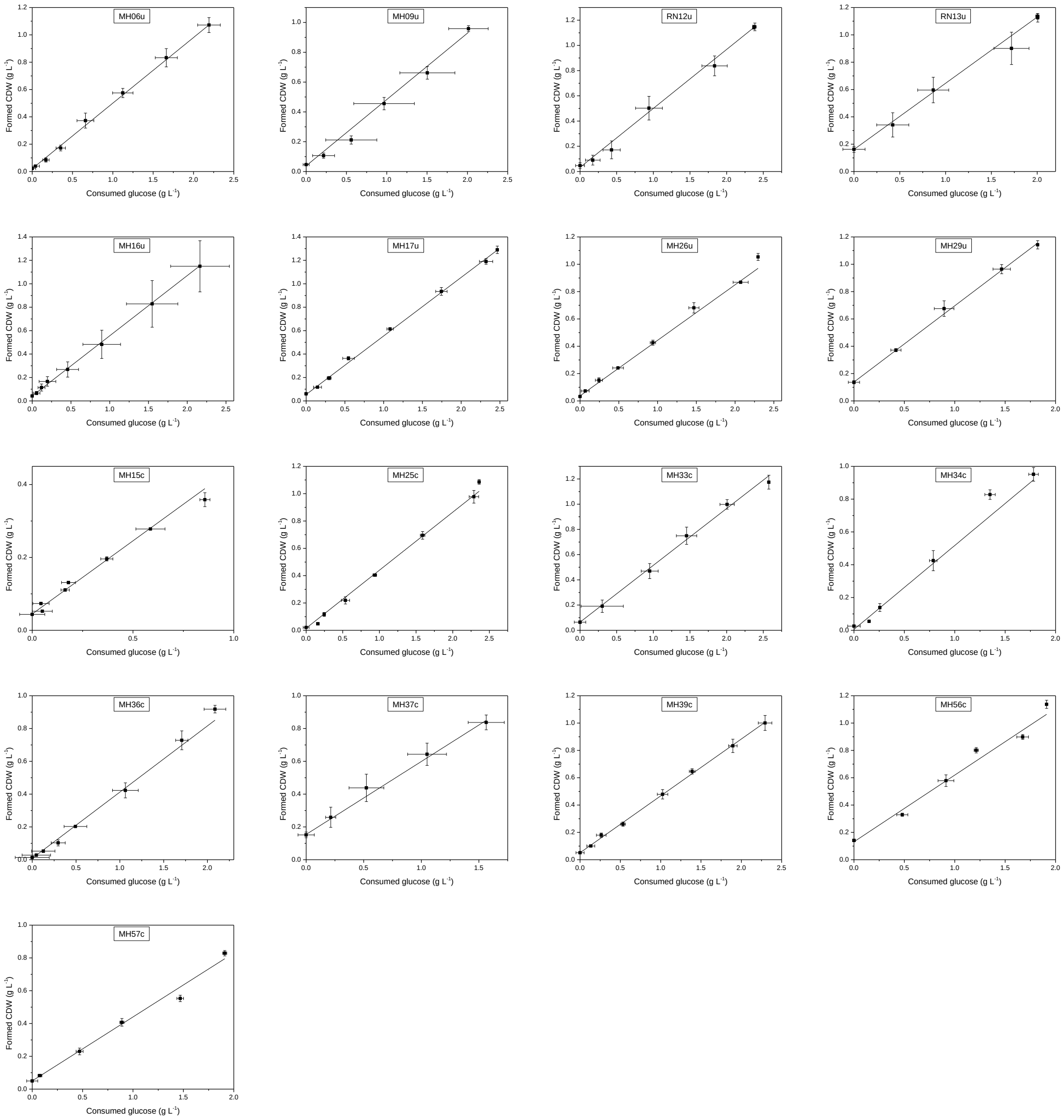

Figure S5. Metabolic profiles of consumed glucose concentration and formed cell dry weight of clinical *P. aeruginosa* isolates on minimal glucose medium (three biological replicates each). Metabolic steady-state is inferred from the constant yield for biomass, derived as slope from the profiles.
