# Supplementary material for: Robustness and Plasticity of Metabolic Pathway Flux among Uropathogenic Isolates of Pseudomonas aeruginosa
Source: PLoS One. 2014 Apr 7;9(4):e88368. doi: 10.1371/journal.pone.0088368 (PMC3977821; doi:10.1371/journal.pone.0088368)
Supplement: Table S1 — Refinement of the cellular composition of P. aeruginosa by quantification of the alginate capsule and the corresponding anabolic demand for F6P for its biosynthesis. For quantification, alginate was detached from the cells, and then analyzed by the sulfamate-biphenyl method [1]. Shortly, cells were harvested, washed once with deionized water. Subsequently, the alginate was detached by shaking for 5 h (300 µL 0.14 M NaCl suspension, 1400 min−1, 25°C). The supernatant (200 µL), obtained by centrifugation (15 min, 16,000× g, 4°C), was then amended with 20 µL 4 M sulfamate and with 1.2 mL 0.075 M tetraborate, dissolved in concentrated H2SO4, and then incubated for 20 min at 99°C. Afterwards, the suspension was transferred to an ice bath (5 min), followed by addition of 40 µL 0.15% 3-hydroxybiphenyl, dissolved in 0.5% NaOH. The mixture was incubated for 10 min at room temperature. Alginate was quantified by photometry at 525 nm, using isolated alginate from mucoid P. aeruginosa FRD1 as external standard [2]. The data reflect the F6P demand for alginate biosynthesis and are given as relative flux, normalized to the specific glucose uptake rate (Table 1). These values were additionally considered in the anabolic requirement for flux analysis by correcting the demand for F6P (Table S2). Generally, the requirement was low. (PDF) [file pone.0088368.s006.pdf]

Table S1: Refinement of the cellular composition of *P. aeruginosa* by quantification of the alginate capsule and the corresponding anabolic demand for F6P for its biosynthesis. For quantification, alginate was detached from the cells, and then analyzed by the sulfamate-biphenyl method [1]. Shortly, cells were harvested, washed once with deionized water. Subsequently, the alginate was detached by shaking for 5 h (300 µL 0.14 M NaCl suspension, 1400 min^{-1}, 25°C). The supernatant (200 µL), obtained by centrifugation (15 min, 16,000x*g*, 4°C), was then amended with 20 µL 4 M sulfamate and with 1.2 mL 0.075 M tetraborate, dissolved in concentrated H_2SO_4, and then incubated for 20 min at 99 °C. Afterwards, the suspension was transferred to an ice bath (5 min), followed by addition of 40 µL 0.15% 3-hydroxybiphenyl, dissolved in 0.5% NaOH. The mixture was incubated for 10 min at room temperature. Alginate was quantified by photometry at 525 nm, using isolated alginate from mucoid *P. aeruginosa* FRD1 as external standard [2]. The data reflect the F6P demand for alginate biosynthesis and are given as relative flux, normalized to the specific glucose uptake rate (Table 1). These values were additionally considered in the anabolic requirement for flux analysis by correcting the demand for F6P (Table S2). Generally, the requirement was rather low.

**Strain**	**Biosynthetic flux (%)**
PAO1	0.00
MH15c	0.16
MH25c	0.00
MH33c	0.35
MH34c	0.00
MH36c	0.00
MH37c	0.35
MH39c	0.38
MH56c	0.00
MH57c	0.16
MH06u	0.35
MH09u	0.00
RN12u	0.58
RN13u	0.47
MH16u	0.16
MH17u	0.00
MH26u	0.28
MH29u	0.16

References

1. Filisetti-Cozzi TM, Carpita NC (1991) Measurement of uronic acids without interference from neutral sugars. Anal Biochem 197: 157–162.
2. Tielen P, Narten M, Rosin N, Biegler I, Haddad I et al. (2011) Genotypic and phenotypic characterization of *Pseudomonas aeruginosa* isolates from urinary tract infections. Int J Med Microbiol 301: 282–292.
